# Features of databases that supported searching for rapid evidence synthesis during COVID-19: implications for future public health emergencies

**DOI:** 10.1186/s12874-024-02246-x

**Published:** 2024-06-21

**Authors:** Leah Hagerman, Emily C. Clark, Sarah E. Neil-Sztramko, Taylor Colangeli, Maureen Dobbins

**Affiliations:** 1 National Collaborating Centre for Methods and Tools, McMaster University, McMaster Innovation Park, 175 Longwood Rd S, Suite 210a, Hamilton, ON L8P 0A1 Canada; 2 Department of Health Research Methods, Evidence & Impact, McMaster University, McMaster University Medical Centre, 2C Area, 1280 Main St W, Hamilton, ON L8S 4K1 Canada; 3 School of Nursing, Health Sciences Centre, McMaster University, 2J20, 1280 Main St W, Hamilton, ON L8S 4K1 Canada

**Keywords:** Rapid reviews, Evidence- informed decision making, Public health, Searching, Databases, Repositories, Systematic reviews, COVID-19

## Abstract

**Background:**

As evidence related to the COVID-19 pandemic surged, databases, platforms, and repositories evolved with features and functions to assist users in promptly finding the most relevant evidence. In response, research synthesis teams adopted novel searching strategies to sift through the vast amount of evidence to synthesize and disseminate the most up-to-date evidence. This paper explores the key database features that facilitated systematic searching for rapid evidence synthesis during the COVID-19 pandemic to inform knowledge management infrastructure during future global health emergencies.

**Methods:**

This paper outlines the features and functions of previously existing and newly created evidence sources routinely searched as part of the NCCMT’s Rapid Evidence Service methods, including databases, platforms, and repositories. Specific functions of each evidence source were assessed as they pertain to searching in the context of a public health emergency, including the topics of indexed citations, the level of evidence of indexed citations, and specific usability features of each evidence source.

**Results:**

Thirteen evidence sources were assessed, of which four were newly created and nine were either pre-existing or adapted from previously existing resources. Evidence sources varied in topics indexed, level of evidence indexed, and specific searching functions.

**Conclusion:**

This paper offers insights into which features enabled systematic searching for the completion of rapid reviews to inform decision makers within 5–10 days. These findings provide guidance for knowledge management strategies and evidence infrastructures during future public health emergencies.

## Introduction

Throughout the Coronavirus Disease 2019 (COVID-19) pandemic, policy- and decision-makers had an unprecedented demand for synthesized evidence, often needing the evidence within hours or days. This rapid process sparked the need to quickly find and assess evidence for relevance [1–4]. However, the vast number of new studies and changing terminology during the COVID-19 pandemic made finding relevant emerging evidence increasingly challenging. While open access availability of articles related to COVID-19 has been invaluable to researchers and decision-makers worldwide, the high volume of new articles and evidence syntheses created a considerable challenge for those conducting rapid reviews in response to decision-maker requests: by June 1, 2020, in PubMed alone, there were 16,670 articles related to the COVID-19 pandemic [5], with the number of both published and preprint articles continuing to surge into November 2020 [5, 6]. Changing terminology posed an additional challenge; for example, until the International Committee on Taxonomy of Viruses and the World Health Organization (WHO) officially named the novel coronavirus the *severe acute respiratory syndrome coronavirus 2 (SARS-CoV-2)* and the associated illness *coronavirus disease (COVID-19)* on February 1st, 2020 [7], many scientific and non-scientific names were used when referring to the virus (e.g., 2019-nCov, Wuhan virus and China virus) [8] and the associated disease (e.g., pneumonia or acute respiratory illness without a known cause) in the literature. Such additional names persisted beyond February 2020 in both the academic and non-academic literature [9], thus impacting synthesis research throughout the summer and fall of 2020.

In response to these challenges, new evidence sources specifically designed to house evidence on SARS-CoV-2 and COVID-19 emerged quickly in 2020 (e.g., PubMed’s LitCovid), while existing evidence sources were modified to capture and categorize this new literature (e.g., the creation of the COVID-19 filter in PROSPERO) [10–14]. These new evidence sources aimed to compile emerging evidence in one location to facilitate access to the most relevant and up-to-date literature for researchers and decision-makers to support evidence-informed decision making [10–12].

To best meet decision-makers’ needs for answers to priority public health questions within short timelines, many organizations turned to rapid review methodology, including the National Collaborating Centre for Methods and Tools (NCCMT) in Canada [1–4]. The NCCMT, along with five additional National Collaborating Centres for Public Health, was created by the Public Health Agency of Canada (PHAC) in 2005 in response to the 2003 Severe Acute Respiratory Syndrome (SARS) epidemic [15]. Together, the National Collaborating Centres for Public Health exist to strengthen public health by supporting the timely use of scientific evidence. The NCCMT’s mission is to provide high-quality resources and real-world training to support the ever-changing needs of public health to improve the health and well-being of every person living in Canada [16–18]. Within weeks of the pandemic being declared, the NCCMT started receiving requests from public health decision-makers in Canada for support in synthesizing the emerging COVID-19 literature. The NCCMT responded by creating a Rapid Evidence Service (RES), establishing processes for accepting requests, refining questions, searching for, appraising and synthesizing evidence, and disseminating the knowledge products. The NCCMT’s goal in creating the RES was to support an evidence-informed response in Canada to the pandemic [17, 19]. A comprehensive description of the RES methods is published [17], including descriptions of how searching was conducted in established evidence sources (e.g., MEDLINE and PROSPERO [13, 20]), evidence sources developed specifically for COVID-19 evidence (e.g., PubMed’s LitCovid and the Oxford COVID-19 Evidence Service [21, 22]), preprint servers (e.g., MedRxiv [23]), and grey literature sources [17]. Evidence sources used by the NCCMT for the RES were chosen based on topic relevance, ease of searching, and continued evidence source maintenance [17].

While the RES team benefited from the newly created covid evidence sources for developing its search strategies for rapid reviews, substantial challenges were still encountered in effectively and efficiently identifying relevant evidence for rapid reviews. These experiences may inform approaches to evidence retrieval and management during future global emergencies. The aim of this paper is to explore the features and functions most essential to supporting systematic searching for emergent public health evidence, and make recommendations for priority features for evidence sources, that will better support evidence synthesis in future public health emergencies. Through this paper, we describe the strengths and limitations of the features and functions of each source and how these influenced the evolution of our rapid review search strategies. Finally, we discuss implications for the development of knowledge management strategies that can respond to emergent situations.

## Methods

The RES methods included searching databases (e.g., Cochrane Library [24]), platforms (e.g., MEDLINE [20]), federated search systems (e.g., the Living OVerview of Evidence (LOVE) [25]) and repositories (e.g., MedRxiv [23]). For the purpose of this paper, all are referred to as “evidence sources,” as all were searched using variations of an advanced keyword string specific to each rapid review topic [17]. To describe and compare evidence sources that were routinely used when completing RES reviews, each searchable evidence source was reviewed by two authors (LH, TC) in August 2021 and checked again for updates by one author (LH) in April 2023. All evidence sources are listed in Table 1. Specific functions of each evidence source, as they pertain to searching in the context of a public health emergency, were recorded based on practical experience and reviewing “About Us”, “Help”, “Frequently Asked Questions” pages and background literature provided on the respective websites.

**Table 1:** Topics, types of evidence, and search features of evidence sources

	Evidence Sources	Description	Topic/Specialty						Level and Type of Evidence			
			IPAC^a^	Disease Characteristics	Surveillance & Epidemiology	Equity	Policy	Mental Health & Substance Use	Syntheses	Single Studies	Preprints	Other^b^
Newly developed	LitCovid [10]	Central database indexing COVID-19 articles from PubMed; updated daily	✓	✓	✓	✓	✓	✓	✓	✓	✕	✓
	LOVE [25]	Repository indexing COVID-19 published studies, preprints, and clinical trials; updated daily	✓	✓	✓	✓	✓	✓	✓	✓	✓	✓
	NCCMT Repository of Public Health Evidence Syntheses [26]	Repository indexing completed and in-progress public health rapid evidence reviews; updated ad hoc	✓	✓	✓	✓	✓	✓	✓	✕	✕	✕
	WHO [14]	Central database indexing COVID-19 articles; updated daily	✓	✓	✓	✓	✓	✓	✓	✓	✓	✓
Pre-established	Cochrane Library [24]	Collection of databases that index health studies; updated daily	✓	✓	✓	✓	✓	✓	✓	✓	✕	✓
	Embase via Ovid [27]	Central database indexing biomedical and pharmacological studies; updated daily	✓	✓	✓	✓	✓	✓	✓	✓	✕	✓
	ERIC via ProQuest [28]	Central database indexing education articles; updated daily	✕	✕	✕	✓	✕	✕	✓	✓	✕	✓
	McMaster PLUS™ [29]	Repository indexing pre-appraised clinical studies; updated daily	✓	✓	✓	✓	✓	✓	✓	✓	✕	✕
	MEDLINE via Ovid [20]	Central database indexing life sciences and biomedical studies; updated daily	✓	✓	✓	✓	✓	✓	✓	✓	✕	✓
	MedRxiv [23]	Health sciences preprint server; updated daily	✓	✓	✓	✓	✓	✓	✓	✓	✓	✓
	PROSPERO [13]	Open access database of systematic review protocols; updated daily	✓	✓	✓	✓	✓	✓	✓	✕	✕	✕
	PsyArXiv [30]	Psychology preprint server; updated daily	✕	✕	✕	✕	✕	✓	✓	✓	✓	✕
	Trip [31]	Search engine indexing various topics; updated daily	✓	✓	✓	✓	✓	✓	✓	✓	✕	✓

	Evidence Sources	Features										
		Advanced Search					Filters		Sort	Export		
		Subject headings	Boolean expressions	Parentheses	Phrase searching	Truncations	By title and/or abstract	By date	Within database	Full list	Partial list	
Newly developed	LitCovid [10]	✕	✓	✓	✓	✕	✕	✕	✓	✓	✕	
	LOVE [25]	✕	✓	✓	✓	✓	✕	✓	✓	✓	✓	
	NCCMT Repository of Public Health Evidence Syntheses [26]	✕	✓	✓	✓	✓	✕	✕	✕	✓	✓	
	WHO [14]	✕	✓	✓	✓	✓	✓	✓	✓	✓	✓	
Pre-established	Cochrane Library [24]	✓	✓	✓	✓	✓	✓	✓	✓	✓	✓	
	Embase via Ovid [27]	✓	✓	✓	✓	✓	✓	✓	✓	✓	✓	
	ERIC via ProQuest [28]	✓	✓	✓	✓	✓	✓	✓	✓	✓	✓	
	McMaster PLUS™ [29]	✕	✓	✓	✓	✓	✕	✓	✓	✓	✓	
	MEDLINE via Ovid [20]	✓	✓	✓	✓	✓	✓	✓	✓	✓	✓	
	MedRxiv [23]	✕	✕	✕	✕	✓	✓	✓	✓	✕	✓	
	PROSPERO [13]	✕	✓	✓	✓	✓	✕	✓	✓	✓	✓	
	PsyArXiv [30]	✕	✓	✓	✓	✓	✕	✕	✓	✕	✕	
	Trip [31]	✕	✓	✓	✓	✓	✓	✓	✓	✓	✓	

✓ = included in the evidence source; ✕ = not included in the evidence source

^a^Infection Prevention and Control

^b^Other types of evidence not typically included in RES reviews, including commentaries, opinion pieces, reports, news articles, recommendations and guidance documents, published protocols, and repositories of raw data

Information was collected across three domains: (1) Topics and specialties of indexed citations, aligned with the categories of questions answered by the RES (i.e., infection prevention and control, disease characteristics, surveillance and epidemiology, equity, policy, and mental health and substance use); (2) Type of evidence indexed (e.g., systematic reviews, single studies, registered protocols, and preprint studies); and (3) Search features (e.g., advanced search functions, search filters, citation exports, and citation sorting). Information collected across data sources is presented descriptively. Through a descriptive analysis, we present similarities and differences across sources, and present features and functions that were found to enhance usability in the context of an evolving public health emergency. Specific usability features that informed methodological decisions for searching for RES rapid reviews are also presented.

## Results

From the RES inception in 2020 to our scan of resources in 2023, a total of thirteen evidence sources were included in the RES. Of these 13 evidence sources, four were newly created in response to the COVID-19 pandemic (LitCovid, LOVE, NCCMT Repository of Public Health Evidence Syntheses, World Health Organization’s (WHO) Global research on coronavirus disease), and nine were either pre-existing or adapted from previously existing resources (Cochrane Library, Embase, ERIC, McMaster PLUS, MEDLINE, MedRxiv, PsyArXiv, PROSPERO, Trip). A summary of all data sources is presented in Table 1.

### Topic/Specialty

The topics and specialties of the evidence sources align with priority topic areas in public health (Table 1). Six priority topic areas were identified, including: Infection Prevention and Control; Disease Characteristics; Surveillance and Epidemiology; Equity; Policy; and Mental Health and Substance Use. Nearly all sources included evidence relevant to all six topic areas, with the exception of ERIC and PsyArXiv. As these evidence sources traditionally have focused on education and psychology literature, respectively, this was expected. There were no notable differences in topics indexed between newly created and pre-existing evidence sources.

### Type of evidence

The RES assessed evidence sources based on level of evidence, i.e., syntheses and single studies, which were further categorized as either peer-reviewed studies, preprints, or registered protocols. Syntheses were indexed in all 13 evidence sources, with single studies indexed in 11 (LitCovid, LOVE, WHO, Cochrane Library, Embase, ERIC, McMaster PLUS, MEDLINE, MedRxiv, PsyArXiv, Trip) [10–12, 14, 20, 23, 24, 27, 28, 30, 31]. Two evidence sources (MedRxiv, PsyArXiv) exclusively housed preprints [23, 30], while two additional evidence sources (LOVE, WHO) indexed preprints as well as published literature [14, 25]. Nine evidence sources (LitCovid, LOVE, WHO, Cochrane Library, Embase, ERIC, MEDLINE, MedRxiv, Trip) included additional types of evidence not typically included in RES reviews, such as expert opinion pieces, guidelines, and ongoing clinical trials [14, 20, 21, 23–25, 27, 28, 31]. Newly established evidence sources more often included preprints and other forms of evidence, such as opinion pieces, guidelines, and ongoing clinical trials whereas existing evidence sources did not. One newly developed evidence source (NCCMT Repository of Public Health Syntheses) exclusively indexed completed and in-progress syntheses [26].

### Advanced features

#### Advanced search

Advanced searching, i.e., the ability to search using subject headings, Boolean expressions, parentheses, phrase searching, and/or truncations, was the most common of the advanced features; all evidence sources (LitCovid, LOVE, NCCMT Repository of Public Health Evidence Syntheses, WHO, Cochrane Library, Embase, ERIC, McMaster PLUS, MEDLINE, MedRxiv, PROSPERO, PsyArXiv, Trip) included at least one advanced search function with no major differences between new and pre-existing evidence sources [13, 14, 20, 21, 23–31]. The advanced search features varied in complexity: four (Cochrane Library, Embase, ERIC, MEDLINE) allowed for subject headings or key term mapping [20, 24, 27, 28], and all but one (MedRxiv) allowed for Boolean expressions, parentheses, phrase searching and truncations [23].

#### Filters

Evidence sources provided various filtering options, including filtering by title and/or abstract and by date. Seven evidence sources (WHO, Cochrane Library, Embase, ERIC, MEDLINE, MedRxiv, Trip) could filter by title and/or abstract and by date [14, 20, 23, 24, 27, 28, 31]; three (LOVE, McMaster PLUS, Prospero) could filter by date only [13, 25, 29]; and three (LitCovid, NCCMT Repository of Public Health Syntheses, PsyArXiv) had no filters [10, 26, 30]. Of note, several evidence sources that included advanced search features and filters were designed in such a way that both features could not be used simultaneously, so performing an advanced search on a filtered set of results was not possible. Eight of nine pre-established evidence sources (Cochrane Library, Embase, ERIC, McMaster PLUS, MEDLINE, MedRxiv, PROSPERO, Trip) offered advanced filters [13, 20, 23, 24, 27–29, 31], whereas only two of four newly developed COVID-19-dedicated evidence sources (LOVE, WHO) offered advanced filters [14, 25].

#### Sort

All but one evidence source (NCCMT Repository of Public Health Evidence Syntheses) allowed for sorting of search results [26]. Pre-existing evidence sources offered more sophisticated sorting within the export, such as by specific publication date rather than by year.

#### Export

All newly developed and eight pre-existing evidence sources (Cochrane, Embase, ERIC, McMaster PLUS, MEDLINE, MedRxiv, PROSPERO, PsyArXiv, Trip) included options to export the complete list of search results in an EndNote-compatible format (e.g., .RIS) [14, 20, 21, 24, 25, 27–29, 31, 32]; while eight evidence sources (Cochrane, Embase, ERIC, McMaster PLUS, MEDLINE, MedRxiv, PROSPERO, Trip) had options to export a partial list of search results by applying either a filter or exporting page-by-page [13, 20, 23, 24, 27–29, 31]. Evidence sources varied in the ease of usability of the export function, such as exporting a single page at a time (WHO, ranging from 25 to 100 results per page) [14, 23], up to 200 results at a time (McMaster PLUS [29]) or exporting up to 2,000 results at a time (Medline [20]). One evidence source (PsyArXiv) did not include an export function [30].

## Discussion

The NCCMT RES adapted their search methods according to the changing evidence ecosystem of COVID-19 research, specific parameters of research questions, and evolving search features of the evidence sources. When the RES was first established in April 2020, the team relied heavily on newly established evidence sources to access the most up-to-date evidence on COVID-19, such as PubMed’s LitCovid [21]. Newly established evidence sources included only evidence specific to the COVID-19 pandemic, thereby circumventing issues for researchers of changing and inconsistent terminology [8, 21, 25]. As the volume of records in newly established evidence sources was relatively low early in the COVID-19 pandemic, hand-searching without sorting and export functions was feasible. Within this context, the most important feature was transparency in the topics and types of evidence included in each evidence source, making it possible to search only those evidence sources that indexed evidence relevant to specific research questions. Additionally, to maintain early pandemic turnaround times of five days, pairing filters for date and indexed types of evidence (e.g., syntheses, single studies, grey literature) allowed for searching for and retrieving the most synthesized and current evidence in an efficient manner [17]. This was of particular importance at the beginning of the rapid review cycle to determine if a recent systematic review or rapid review on a given topic already existed, as well as for rapid review updates to determine the types of evidence that had emerged since the last search date. It is recommended that evidence source developers include searching functions that allow researchers to additively filter by topic, date, and type of evidence.

As the pandemic continued and the volume of research literature grew throughout 2020 and 2021, the RES prioritized features that facilitated efficient evidence retrieval and screening. For this, advanced searching features, filters, and sorting, as well as export functions in an EndNote-compatible format (e.g., .RIS) were found to be the most critical for efficiently conducting a rapid review search, whereas controlled vocabulary supported the quick identification of the most relevant evidence and exclusion of non-relevant evidence. We recommend that advanced searching features be embedded within all evidence sources to facilitate searching. Likewise, the ability to sort and filter evidence by title and abstract provided an option to reduce the search volume when needed. Finally, as the language used to describe the COVID-19 pandemic became more consistent across publications, searching in pre-existing evidence sources was prioritized as these generally had the most sophisticated and consistent searching functions, thus streamlining the searching process. Today, as the RES continues to expand beyond COVID-19 literature, searching methods primarily focus on pre-existing evidence sources as these sources capture a broad selection of topics.

Pre-existing evidence sources and those created in response to the COVID-19 pandemic differed in content, usability and functions. Overall, the evidence sources explored here indexed topic-relevant citations, most included a mix of synthesized and single studies, and most included a selection of advanced searching features. In comparing pre-existing evidence sources to those created in response to the COVID-19 pandemic, content and functionality were similar. One notable difference between pre-existing and some COVID-19-dedicated evidence sources is the inclusion of preprints [25]: preprints have, by definition, not undergone peer review, nor have they been accepted for publication in any journal [33]. However, preprints offered the quickest means for newly emerging evidence to be disseminated and made accessible to others [34, 35]. Preprint servers have been widely used to disseminate information throughout the COVID-19 pandemic and host almost 25% of COVID-19-related science, allowing data and findings from preprint articles to be shared across multiple online platforms [35]. Access to unreviewed manuscripts on preprint servers allowed new research to be quickly disseminated to the broader scientific community and facilitated collaborations between teams [36]. Thus, the RES prioritized searching preprint repositories, as well as evidence sources that included preprints as part of their results, for all COVID-19 rapid reviews [23, 30]. However, preprint repositories presented many challenges, namely, systematic reviews that include unreviewed data risk disseminating incorrect or misinterpreted data [36]. While peer review has limits, such as the potential to inhibit innovation and susceptibility to plagiarism, peer review remains a trusted method of sharing and disseminating new scientific findings [37]. The RES therefore used evidence from preprint papers with caution. An additional challenge arose in that, once a relevant preprint had been identified and was ready for data extraction, it was often challenging to determine if a preprint manuscript had been accepted for publication in a peer-reviewed journal. Finally, preprint repositories often lacked advanced search features, creating a potential barrier to their use. To expedite the searching and dissemination of the most current evidence in a public health emergency, preprint servers must provide searching features comparable to evidence sources that index peer-reviewed literature.

Complementary work has been completed by the Library Reserves Corps and their series of recommendations on Best Practices for Searching During Public Health Emergencies [38]. The RES’s methods align with these recommendations, including searching both traditional and emerging sources of evidence and using various sources to capture the latest terminology [38]. To achieve this, it was especially valuable for evidence sources to be transparent about their methods. Additional complimentary work was completed by Gusenbauer and Haddaway, who reviewed 28 search systems to assess the content coverage and capability to perform systematic searches to support researchers in determining the precision, efficiency, and ultimate usability of various evidence sources [39]. The work of Gusenbauer and Haddaway created awareness of search requirements of evidence syntheses among database providers [39]. The current findings expand on the work of Gusenbauer and Haddaway: by understanding the terminology and parameters used by each evidence source, the RES could tailor their search strategies to use the most sophisticated evidence sources available. It is recommended that all evidence sources include transparent methods that clearly outline the parameters of what evidence is captured and indexed.

### Implications & recommendations

The most crucial knowledge management functions were advanced searching functions, such as controlled vocabulary to narrow down a search, and export functions, to allow deduplication of results across evidence sources. Importantly, it was very helpful when specialized evidence sources were transparent about their searching and indexing methods which facilitated coordination of our search strategies across multiple databases. Knowledge of the functions and parameters of each evidence source allowed rapid review searches to be completed more quickly, contributing to maintaining a 5–10-day completion period for each rapid review early in the pandemic, which lengthened to three or more weeks starting in 2021 as the volume of studies for COVID-19 grew and the RES responded to more complex questions. These experiences can help in the development of new evidence sources, enhancement of pre-established evidence sources, and creation of new evidence sources in times of crisis. These findings highlight the need for researchers and database developers to remain flexible while conducting research during times of emergency.

### Limitations

This paper does not provide an assessment of all evidence sources available during and beyond the COVID-19 pandemic, nor does it provide detail of how evidence sources changed and evolved over time; rather, the focus is on evidence sources that were found to be useful in conducting rapid reviews of emergent public health evidence based on a specific point in time. There is potential for these learnings to be applied to new settings, particularly as new knowledge management needs arise during future global emergencies. It is important to note that, as with all rapid reviews, there is a trade-off between speed and rigour, where rapid review teams prioritize finding the most relevant evidence by optimizing both the sensitivity and precision of the search [40]. It is therefore possible that by not searching all available evidence sources, some relevant evidence could have been missed [40].

This paper focused on the public health field. The core evidence sources searched would likely differ for topic areas outside of public health. Still, we believe the processes and implications outlined here regarding searching functions likely apply across all fields. Clinical trials are generally not possible for many public health topics. Therefore, this paper did not explore evidence sources that focus on clinical trials in depth.

## Conclusion

This paper explored the content and functions of key evidence sources that facilitated the rapid synthesis of evidence for decision makers during a global public health emergency. The benefits and limitations of new and pre-existing sources that indexed COVID-19 evidence were explored. Critical features that enabled rapid systematic searching of evidence include sophisticated searching functions and the ability to export results. These findings can help inform the development of new evidence sources and rapid review searching methods in the context of public health emergencies.

## Data Availability

All data generated or analyzed during this study are included in this published article.

## Abbreviations

COVID-19: Coronavirus Disease 2019
ERIC: Education Resources Information Centre
LOVE: Living OVerview of Evidence
NCCMT: National Collaborating Centre for Methods and Tools
PHAC: Public Health Agency of Canada
PRISMA: Preferred Reporting Items for Systematic Reviews and Meta-Analyses
RES: Rapid Evidence Service
SARS: Severe Acute Respiratory Syndrome
SARS-CoV-2: Severe Acute Respiratory Syndrome Coronavirus 2
WHO: World Health Organization
